# Novel Statistical Classification Model of Type 2 Diabetes Mellitus Patients for Tailor-made Prevention Using Data Mining Algorithm

**DOI:** 10.2188/jea.12.243

**Published:** 2007-11-30

**Authors:** Koichi Miyaki, Izumi Takei, Kenji Watanabe, Hiroshi Nakashima, Kiyoaki Watanabe, Kazuyuki Omae

**Affiliations:** 1 Department of Preventive Medicine and Public Health, School of Medicine, Keio University, Tokyo, Japan.; 2 Department of Internal Medicine, School of Medicine, Keio University, Tokyo, Japan.; 3 Department of Oriental Medicine, School of Medicine, Keio University, Tokyo, Japan.; 4 Department of Laboratory Medicine, School of Medicine, Keio University, Tokyo, Japan.

**Keywords:** type 2 diabetes, complications, data mining, classification and regression trees, tailor-made prevention

## Abstract

To estimate the usefulness of data mining algorithms for extracting risk predictors of diabetic vascular complications in proper order in the future, we tried applying the Classification and Regression Trees (CART) method to the prevalence data of 165 type 2 diabetic outpatients and already known risk factors. Among the 6 categorical and 15 continuous risk factors, age (cutoff: 65.4) was the best predictor for classifying patients into groups with and without macroangiopathy (p=0.000). Body weight (cutoff: 53.9) was the best predictor (p=0.006) in the older group (age >65.4), whereas systolic blood pressure (cutoff: 144.5) was the best predictor in the remaining group (p=0.002). Age (cutoff: 64.8) was also the best predictor for categorizing them into groups with and without microangiopathy (p=0.000). In the older group (age >64.8), BMI (cutoff: 21.5) was the best predictor (p=0.001), whereas morbidity term (cutoff: 15.5) was the best predictor in the other group (p=0.010). Because the orders and values of all risk factors and cutoff points mined were reasonable clinically, this method may have the potential to highlight predictors in order of importance to apply tailor-made prevention of diabetic vascular complications.

## INTRODUCTION

Type 2 diabetes mellitus is one of the most common diseases in developed countries. Since its morbidity term is quite long and it greatly affects patient’s quality of life, the prevention of its complications is very important. Various risk factors for diabetic complications have been revealed in previous studies^1^^-^^4^^)^. Age, high blood pressure, long morbidity term, poor glycemic control, high triglyceride, high total cholesterol, high low density lipoprotein, low high density lipoprotein, high BMI, smoking, and male sex are known to be the risk factors for macroangiopathy. Age, poor glycemic control, high blood pressure, high BMI, smoking and male sex are known to be the risk factors for microangiopathy.

Logistic regression analysis in large scale randomized controlled trials has made great contributions to identifying multiple risk factors^5^^-^^7^^)^. It has yielded odds ratios with 95% confidence intervals for each factor, and the bigger the odds ratio is, the larger its influence would be expected to be. However, since the regression model is optimized for fitting to whole samples, this reasoning is valid only in whole populations, not in the latent subgroups within it. Usual statistical methods are not effective enough to indicate the priority of risk factors in individual patients.

Our final goal is to determine which factors contribute most to the occurrence of complications in each latent subgroup by applying the Classification and Regression Trees (CART) method. As a preliminary step, we estimate here the usefulness of this data mining algorithm using the prevalence of diabetic complications and its risk factors which are already known.

## SUBJECTS AND METHODS

We enrolled 165 type 2 diabetic patients from the Keio University Hospital outpatient clinic in the first half of 2000. We explained the study purpose and plan, and got consents by all of them before the enrollment. To protect the privacies, we omitted patient’s name and patient’s number from the data before the analysis in case of data leakage. We took the patients’ past histories of macroangiopathy and microangiopathy as the endpoints of this analysis. Macroangiopathy here means myocardial infarction, coronary stenosis of more than 75% in diameter as confirmed by coronary angiography, cerebral infarction confirmed by either brain magnetic resonance imaging or computed tomography, or a history of amputation. Microangiopathy here means stage progressions of diabetic retinopathy and nephropathy. The stage of retinopathy was diagnosed by ophthalmologists, and patients diagnosed as having diabetic retinopathy were counted. The stage of nephropathy was decided by the urine albumin-creatinine ratio (Alb/Cr) value, and a value of more than 30 mg/gCr was counted, since the Alb/Cr value have been identified as reliable and valid means of estimating albumin excretion rate in clinical settings^8^^,^^9^^)^.

We collected the patients’ clinical and biochemical data and medical histories related to type 2 diabetes, obtaining data on 6 categorical variables (i.e., sex, smoking habit, family histories of coronary artery disease, cerebrovascular disease, hypertension, and type 2 diabetes mellitus), and 15 continuous ones (i.e., age, morbidity term, systolic blood pressure, diastolic blood pressure, body weight, height, body mass index (BMI), maximum weight, hemoglobin A1c, fast blood sugar, total cholesterol, triglyceride, free fatty acid, low density lipoprotein, and high density lipoprotein) as predictor variables for the analysis. All of the measurements were done in the same laboratory at Keio University Hospital. The characteristics of the patients recruited are shown in Table 1.

**Table 1.** Baseline characteristics of patients recruited.

	All patients	With macroangiopathy	Without macroangiopathy	With microangiopathy	Without microangiopathy
	(n=165)	(n=55)	(n=110)	(n=70)	(n=95)
Demographic					
Age (years)*	60.4 (7.28)	64.2 (4.26)	58.5 (7.7)	61.5 (7.85)	59.5 (6.74)
Male/Female	96/69	35/20	61/49	43/27	18/22

Clinical					
Weight (kg)*	60.0 (9.76)	61.7 (8.98)	59.2 (10.1)	60.6 (9.93)	59.6 (9.67)
Height (cm)*	161.4 (8.11)	161.8 (7.09)	161.2 (8.61)	161.7 (8.54)	161.1 (7.82)
Body-mass index (kg/m2)*	22.8 (2.68)	23.7 (2.64)	22.4 (2.61)	23.3 (2.68)	22.4 (2.64)
Maximum weight (kg)*	69.1 (11.1)	70.8 (10.3)	68.2 (11.4)	70.6 (12.1)	67.9 (10.2)
Systolic blood pressure (mmHg)*	136.7 (16.3)	142.6 (16.5)	133.8 (15.5)	137.0 (18.7)	136.5 (14.4)
Diastolic blood pressure (mmHg)*	80.3 (9.70)	82.0 (8.25)	79.5 (10.3)	80.0 (8.45)	80.6 (10.6)
Smoking (%) never/ex or current	60.6/39.4	60.0/40.0	60.9/39.1	64.3/35.7	57.9/42.1
Morbidity term (years)#	9.92 (2-42)	10.6 (3-37)	9.61 (2-42)	10.3 (2-42)	9.64 (2-34)
Family history of cardiac artery disease yes/no	35/130	16/39	19/91	16/54	19/76
Family history of cerebellar vein disease yes/no	50/115	21/34	29/81	22/48	28/67
Family history of hypertension yes/no	71/94	23/32	48/62	29/41	42/53
Family history of diabetes yes/no	88/77	28/27	60/50	39/31	49/46

Biochemical					
Hemoglobin A1c (%)*	7.22 (1.06)	7.30 (1.11)	7.18 (1.04)	7.47 (1.05)	7.04 (1.04)
Fast blood sugar (mg/dl)*	155.4 (40.1)	156.1 (39.9)	155.0 (40.5)	161.6 (40.7)	150.8 (39.3)
Total cholesterol (mg/dl)*	200.0 (32.7)	203.9 (31.4)	198.1 (33.4)	199.9 (32.2)	200.4 (33.3)
Triglyceride (mg/dl)#	96.3 (32-335)	104.1 (38-272)	92.3 (32-335)	95.6 (32-324)	96.8 (32-335)
Free fatty acid (mEq/l)#	0.43 (0.10-1.36)	0.40 (0.10-0.74)	0.46 (0.12-1.36)	0.43 (0.10-1.36)	0.46 (0.14-1.16)
Low density lipoprotein (mg/dl)*	75.8 (12.7)	76.9 (13.1)	75.4 (12.7)	74.2 (13.5)	76.9 (11.9)
High density lipoprotein (mg/dl)*	58.8 (15.3)	57.2 (12.7)	59.6 (16.5)	60.0 (14.6)	57.8 (15.9)

*mean (SD), #geometric mean (range)

**Figure 1.**
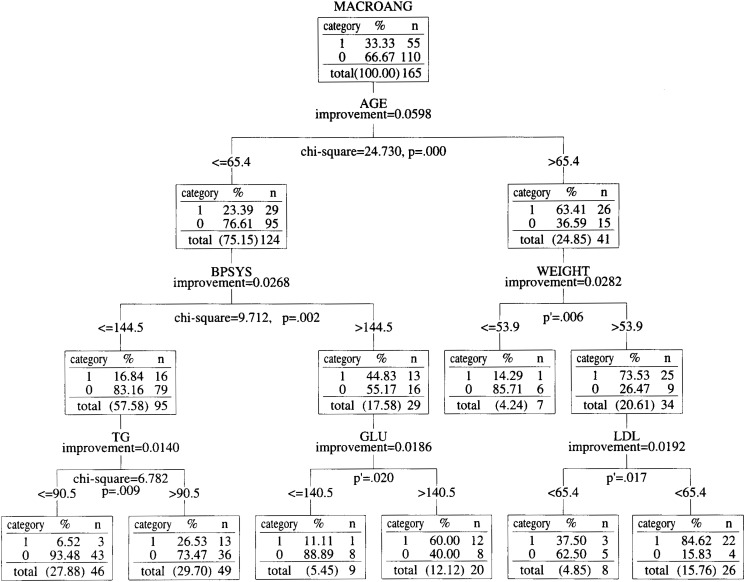
Classification tree of diabetes patients by macroangiopathy risk factors. The root node of Figure 1 indicates that there were 165 total observations, and 55 of the 165 patients have had an episode of macroangiopathy, (i.e., patients belonging to category 1 had a previous episode, and patients belonging to category 0 had not had an episode.) Each node below the root node has the same meaning: the column in the node labeled “category” tells us which category is described in a row, the column labeled “%” tells us what percentage of the subjects in that node fall into that category, and the column labeled “n” tells us how many patients are in that category. At each branch point, “improvement” shows the value of the Gini standard function (see Appendix) at the point, “p” stands for the p value of the chi-square test and “p′ (p prime)” stands for the p value of Fisher’s exact test. All differences are significant at p<0.02. MACROANG, BPSYS, TG, GLU, LDL and FFA in Figure 1 stand for macroangiopathy, systolic blood pressure, triglyceride, fast blood sugar, low density lipoprotein and free fatty acid, respectively.

**Figure 2.**
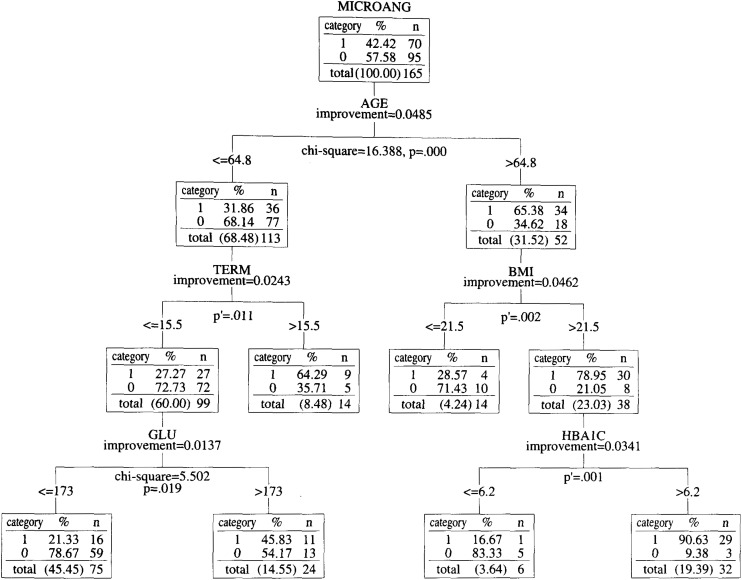
Classification tree of diabetes patients by microangiopathy risk factors. The root node of Figure 2 indicates that there are 165 total observations, and 70 of the 165 patients have had an episode of microangiopathy. The meaning of the nodes in Figure 2 is the same as that in Figure 1. At each branch point, “improvement” shows the value of the Gini standard function (see Appendix) at the point, “p” stands for the p value of the chi-square test and “p′ (p prime)” stands for the p value of Fisher’s exact test. All differences are significant at p<0.02. MICROANG, TERM, BMI, HDL, HBA1C, and GLU in Figure 2 stand for microangiopathy, morbidity term, body mass index, high density lipoprotein, hemoglobin A1c and fast blood sugar, respectively.

We used the Answer Tree 2.1 software produced by the SPSS Company on a Windows NT workstation, and classified the 165 patients by one of the classification tree algorithms called CART. A classification tree is an empirical rule for predicting the class of an object from the values of predictor variables. The concept of classification trees is statistically mature. The first classification tree method was developed by Kass^10^^)^ in 1980 to predict and classify data that were related in complex ways. While this method (the Chi-square Automatic Interaction Detection, CHAID) handles categorical dependent variables, the CART developed by Breiman et al.^11^^)^ in 1984 can also handle continuous variables. CART is a non-parametric method that splits a node based on a data-defined impurity function (i.e., Gini criterion function described in the Appendix). More recently, in 1997, Loh and Shih developed the Quick Unbiased Efficient Statistical Tree (QUEST)^12^^)^, which is intended to be faster computationally and less biased in variable selection than either CHAID or CART. However, QUEST currently handles only categorical dependent variables, and empirical studies indicate that QUEST is not always faster than CART.^13^^)^ Thus, CART remains a popular classification tree method, and that is why we adopted it in this study. The CART method generates binary decision trees. The trees are constructed by splitting subsets of the data set, using all predictor variables to create two child nodes repeatedly, beginning with the entire data set. The best predictor is chosen using impurity measurement (see Appendix). The goal is to produce subsets of data that are as homogeneous as possible with respect to the target variable (i.e., to divide data into high risk groups and low risk groups as clearly as possible). Each predictor is evaluated for each split to identify the best cut point (continuous predictors) or groupings of categories (nominal and ordinal predictors) based on the improvement score (calculated by Gini criterion function defined as in the Appendix) shown at each branch point of the trees (Figures 1 and 2). The predictors are then compared, and the predictor with the best improvement is selected for the split. The process repeats recursively until one of the stopping rules (shown in the Appendix) is triggered^12^^)^. The nearer to the root node, the larger the influence is in regard to classifying patients with or without macroangiopathy and microangiopathy.

At each branch point, we additionally showed the Chi-square value and p value in order to evaluate the dependency of the classification conventionally. In the branch points in which the expected value of at least one cell of 2x2 cells was less than 5, Fisher’s exact test was performed (shown as the p′ value).

## RESULTS

The root node of Figure 1 indicates that 55 of the 165 patients have had an episode of macroangiopathy. Each node below the root node has the same meaning: the column in the node labeled “category,” “%,” and “n” indicate which category is described in a row, what percent of the subjects in that node fall into that category, and how many patients are in the category.

From the standpoint of macroangiopathy, age (cutoff: 65.4) was the best predictor for classifying patients with or without macroangiopathy (p=0.000). In this old (age >65.4) group, body weight (cutoff: 53.9) was the best predictor (p<0.006), whereas systolic blood pressure (cutoff: 144.5) was the best predictor in the other (age <=65.4) group (p<0.002)(Figure 1).

The root node of Figure 2 indicates that 70 of the 165 patients have the episode of microangiopathy. From the standpoint of microangiopathy, aging (cutoff: 64.8) was the best predictor for classifying patients with or without microangiopathy (p=0.000). In this old (age >64.8) group, BMI (cutoff: 21.5) was the best predictor (p<0.001), whereas morbidity term (cutoff: 15.5) was the one in the other (age <=64.8) group (p<0.010)(Figure 2).

In the same way, each node of the tree diagram is interpreted, and represents the patient categories. Due to the recursiveness of the CART algorithm, we can find which factors principally contribute to the occurrence of the complications in each category. For example, from the standpoint of macroangiopathy risk, patients who belong to the old (age >65.4) and heavy (weight >53.9) group in Figure 1 should pay more attention to lowering their LDL level, since it is the most dominant factor.

All the classifications were statistically significant at the 0.02 criterion level (two-sided) by the Chi-square test or Fisher’s exact test.

## DISCUSSION

The cutoff values derived from the CART algorithm seem consistent with clinical experiences. The cutoff value for systolic blood pressure (144.5) is very close to the WHO’s criterion for hypertension (i.e., 140). The cutoff value for fast blood sugar (140.5) is about the same as the old criterion for the diagnosis of diabetes of the American Diabetes Association (i.e., 140). All the other cutoff values were also reasonable clinically. Age, blood pressure, weight control, glycemic control and morbidity term are the dominant risk factors clinicians of the authors predicted before the analysis. The mined cutoff value of age (about 65 in both tree models) is not only near to the predicted value, but also to the administrative senescent cutoff (65 in Japan). Additional Chi-square tests and Fisher’s exact tests showed that all of the classifications were statistically significant. These results indicate that this classification method efficiently categorizes diabetic patients with or without diabetic complications to some extent.

Examination of the nodes one by one shows that the most dominant factor differs with the patient category. The results indicate that the risk factors which type 2 diabetic patients must focus on have an order of priority, and that they differ with patient category. For example, from the standpoint of macroangiopathy risk, patients who belong to the middle-aged (age <=65.4) and hypertensive (systolic blood pressure >144.5) group in Figure 1 should pay more attention to lowering their fast blood sugar. However, patients who belong to middle-aged and normotensive group in Figure 1, should pay more attention to lowering their triglyceride level.

Patients at diabetes outpatient clinics often receive many instructions from their doctors on their lifestyles and day-to-day treatment. It can be difficult for the patients to sort out and follow all of their doctors’ directions at the same time. It would be less confusing for the patients if these directions were given in order of priority, preferably in written form. Educational materials for diabetic patients illustrating the order of priority of treatment would help patients to more easily follow their doctors’ advices. This study presents the possibility that the classification tree method could present the evidence needed to determine an order of priority of clinical treatments and directions concerning risk factors, which could ultimately reduce the complications associated with this disease.

Since most of the predictive variables of patients in this study were collected cross-sectionally after the occurrence of the complications, we cannot insist upon the causal relations between the predictors and the outcomes. This study only describes the prevalence of macroangiopathy or microangiopathy as varying with patient’s characteristics, and that the factors have an order in terms of influence.

Because the CART algorithm was originally designed for large-scale samples, the greater the enrolled number, the more the classification tree will grow, and the more useful the knowledge it produces will be.

We next plan to increase the number of cases and change the study design. We are now engaged in a retrospective cohort study with more than four times of the cases to estimate the occurrence of macroangiopathy or microangiopathy from predictive variables.

If used properly with an appropriate study design, this data mining method may have the potential to highlight predictors in order of importance, thus leading to tailor-made prevention plans for individual patients.

## Appendix

CART stands for Classification and Regression Trees. It is a binary tree growing algorithm developed by Breiman, Friedman, Olshen, and Stone^9^^)^.

CART partitions the data into two subsets so that the cases within each subset are more homogeneous than in the previous subset.

The split of the node in the CART algorithm is determined by the idea of impurity.

Impurity (i.e., the Gini index) at node t, g(t), is defined as

g(t)=∑(j not= i)p(j|t)p(i|t),

where i and j are categories of the target variable, and p means the proportion.

When the cases in a node are evenly distributed across the categories, the Gini index takes its maximum value of 1-1/k, where k is the number of categories for the target variable. When all cases in the node belong to the same category, the Gini index equals 0.

Using this index, the improvement score (i.e., Gini standard function) at node t, f(t), is defined as

$$f(t)=g(t)-P_{\text{L}}\;g(t_{\text{L}})-P_{\text{R}}\;g(t_{\text{R}}),$$

where P_L_ is the proportion of cases in t sent to the left child node, and P_R_ is the proportion sent to the right child node.

The cutoff values of many variables are tried systematically and the improvement scores are calculated in every trial. The split is chosen at the cutoff value under which condition the score is maximum.

The process is repeated recursively until one of the following stopping rules is triggered.

The stopping rules:

The following conditions will cause the algorithm to terminate:

1) The maximum tree depth has been reached.

2) No more splits can be made, because all terminal nodes meet one or more of the following conditions:

a) There is no significant predictor variable left to split the node.

b) The number of cases in the terminal node is less than the minimum number of cases for parent nodes.

c) If the node were split, the number of cases in one or more child nodes would be less than the minimum number of cases for child
